# Including patient and public contributors on clinical trial Independent Data Monitoring Committees (IDMCs)

**DOI:** 10.1186/s13063-026-09559-w

**Published:** 2026-02-17

**Authors:** Elizabeth Allaway, Irene Soulsby, Nicole Keyworth, Louise Stanton, Geoff Saunders, Joshua Caddy, Cherish Boxall, Gareth Griffiths

**Affiliations:** 1 Southampton Clinical Trials Unit, University of Southampton and University Hospital Southampton NHS Foundation Trust, Southampton, UK; 2 Public Contributor, C/O Southampton Clinical Trials Unit, Southampton, UK

**Keywords:** Patient and public involvement (PPI), Public contributor, Independent Data Monitoring Committees (IDMC), Clinical trials, Training

## Abstract

**Background and aims:**

Patient and public involvement (PPI) is well established in clinical trials and trial oversight groups in the UK. But public contributors are rarely involved in Independent Data Monitoring Committees (IDMCs), meaning there is little or no evidence for how to include the public in these complex meetings or the impact PPI may have. As a Cancer Research UK core-funded clinical trials unit, the Southampton Clinical Trials Unit (SCTU) coordinates numerous phase II and III cancer drug trials with IDMCs. We aimed to establish a process to include PPI in these committees, but first needed to assess the feasibility of including public contributors and determine what training and resources they need to take part. Here we summarise our process of developing these training and resources to inform others who wish to include public contributors onto trial IDMCs.

**Methods and findings:**

We used design-based and action research methods to develop an initial training programme, working with trial managers and statisticians at the SCTU to ensure key elements of the IDMC process were covered and explained. This was piloted in a SCTU cancer trial, and feedback from the public contributor, trial staff, Chief Investigator and IDMC chair was used to refine the process. The programme was then further evaluated in three more SCTU trials, and feedback was again gathered through participant questionnaires. Our findings show that the training was useful and informative for public contributors, allowing them to contribute meaningfully to IDMC meetings, while constructive feedback allowed us to refine the process further for future roll-out across SCTU trials.

**Conclusions:**

The findings of this project allowed us to develop, review and refine the training materials and resources required for public contributors to meaningfully take part in IDMCs. Furthermore, feedback received from public contributors, trial teams and IDMC chairs was largely positive and suggests that the inclusion of public contributors, when adequate training and resources are provided, is feasible, can enhance the work of these committees and is welcomed by those involved. Limitations to the research and future work have been identified to further assess the impact of including public contributors in IDMCs.

**Supplementary Information:**

The online version contains supplementary material available at 10.1186/s13063-026-09559-w.

## Background

Patient and public involvement (PPI) is defined by the UK National Institute for Health and Care Research (NIHR) as “research being carried out ‘with’ or ‘by’ members of the public rather than ‘to’, ‘about’ or ‘for’ them” [1]. PPI is well established in the field of clinical trials in the UK [2] where the inclusion of public contributors, people with a direct lived experience of a health condition or experience of caring for someone with a condition, can help to ensure that the research being conducted is relevant to the patient population and may ultimately result in patient benefit such as the treatment arm of a clinical trial becoming a future standard of care in the NHS.

Currently, the network of UK Clinical Research Collaboration Clinical Trials Units (UKCRC CTUs) routinely involves public contributors in its clinical trials as it helps improve trial design, ensuring that the procedures and expectations within a clinical trial are acceptable to patients, and ensuring relevant outcome measures [3]. This includes (i) the early development of the trial concept, design and justification; (ii) the development of the funding bid (with public contributors increasingly included as co-applicants); (iii) when trials are funded (by funding committees that include public contributors) the trial management group (TMG) will include public members who are involved in the set-up and day-to-day management and delivery of the trials; and (iv) increasingly the interpretation and write up of the results and as co-authors on the resulting publications [4].

Public contributors are also increasingly involved within wider CTU processes and committees, such as: CTU Trial Review Groups where decisions are made whether a CTU will support a trial proposal from the clinical community; as independent members of PPI groups who peer review proposed trial materials (e.g. Southampton CTU has the Southern Cancer Trials Public Involvement Group where public contributors peer review trial proposals, protocols and participant information leaflets before they are submitted to the regulatory authorities); on funding committees, for example Cancer Research UK has included public contributors on all its trial funding committees for many years; on independent Trial Steering Committees (TSCs) [5] which act as the executive group on behalf of the trial sponsor to oversee the running of a clinical trial and can recommend actions or even close trials down and report early; and Data Sharing Committees, where decisions are made about how and when to share trial samples and data with other researchers, in accordance with a trial’s specific participant consent forms. Many CTUs also include public contributors on methodology projects to improve clinical trial conduct, for example, in areas such as how to develop and deliver trials that increase recruitment from underserved communities and increase retention.

Southampton CTU (SCTU), a Cancer Research UK core funded CTU and part of the newly formed NIHR Research Support Service, has PPI representation on all the above. However, on reviewing its PPI strategy in 2022, it became clear there was one CTU committee that does not routinely include public contributors, and that is the Independent Data Monitoring Committee (IDMC), sometimes referred to as Data Monitoring and Ethics Committee (DMEC) or Data Monitoring and Safety Committee (DMSC). IDMCs are made up of independent members (clinicians and statisticians) and non-independent members (CI, trial managers (TMs)). The independent members are often the only group who see the results of the trial while it is running and are required to consider risk factors involved in a study, monitor live trial data, and ensure the wellbeing and safety of study participants [6, 7]. They then make recommendations to the TSC about whether the trial should continue, be modified or even stopped, given the data they have observed and considering the safety to trial participants, early signs of benefit or detriment to the treatment arm under investigation and viability of a study [8, 9].

Although there has been some discussion on the inclusion of public contributors in IDMCs [10], and the MRC Clinical Trials Unit at UCL has created a PPI induction pack that includes information for PPI contributors involved with IDMCs [11], the absence of public contributors on IDMCs is the norm for UKCRC CTUs. This is mainly due to the potential misconceptions that public members are not interested in these aspects of trials, that these aspects are too difficult to communicate effectively, and being unsure when public contributors should be included [10]. Staley [3] concludes that this lack of involvement means there is little or no evidence for the impact PPI may have in quantitative data analysis.

Cobb and Moore [12] highlight the role of context in data analysis—“data are not just numbers, they are numbers with a context” and lay public contributors with lived experience may play an important role in providing this context [13]. If patient wellbeing and safety are being discussed on IDMCs, there is a logical justification that there should be a patient voice on a committee with this responsibility and oversight. There is also some evidence to show that patient and public partners are interested in being involved in the more numerical aspects of research and data analysis and see this as important for increasing transparency in clinical trials, but that more support is needed to allow them to do this [10].

However, embedding PPI in research and clinical trials can be complex and resource intensive, often with no extra time or resources allocated to this task [14]. For PPI in data-driven settings such as IDMCs, there is likely to be a requirement for more data-specific training, similar to that suggested as professional training for other IDMC members [9] but tailored to public contributors, and increased support for public contributors who may have little knowledge of these areas [15].

Initial discussions with trial teams, statisticians and directors at SCTU, including clinicians, indicated that there is a need to explore the role of PPI on IDMCs and led to the development of this project. Our aim was to establish PPI representation on SCTU trial IDMCs. This study was designed to assess the feasibility of including public contributors and establish what training and resources they need to take part in these meetings.

## Methods

The project used the principles of design-based research and action research [16], which use participatory research methods to interact and collaborate with stakeholders in an iterative design, feedback and refinement process to identify problems within systems and develop potential solutions and resources [17, 18].

**Fig. 1:**
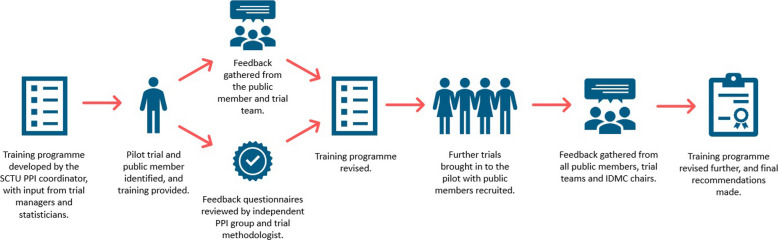
Schema for the SCTU IDMC pilot project

The project was led and facilitated by the SCTU PPI coordinator, with the project team including TMs, statisticians, a mixed methodologist and public contributors (2 female and 2 male). The project included (i) development of a training programme for including public contributors on an IDMC; (ii) evaluation of the training programme using a SCTU pilot trial; (iii) refinement of the training programme using feedback from the pilot trial and independent peer review by a PPI group; (iv) further evaluation in an additional 3 SCTU trials; and (v) using feedback to finalise the training programme for future trials (Fig. 1).

### i) Development of a process for including a public contributor on an IDMC

**Fig. 2:**
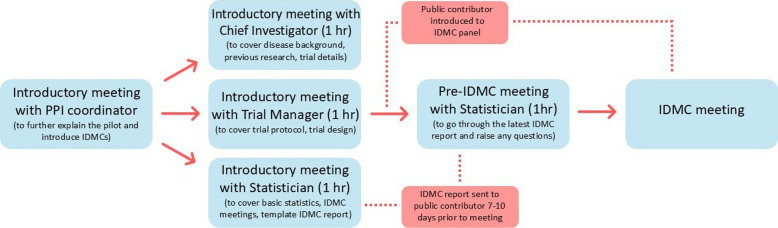
Initial training programme for public contributors on IDMCs

The SCTU PPI coordinator, working with TMs and statisticians, devised an initial project plan and training programme for public contributors in order to provide the necessary support for them to be able to understand and contribute to the IDMC meetings (Fig. 2). This involved reviewing the current PPI training already provided at SCTU, the complexities and demands of IDMCs, and potential knowledge gaps that public contributors may have, and discussing what information would need to be provided and the format this should take. Although more time consuming, it was decided that three separate introductory meetings covering distinct areas of the trial, the IDMC and statistical background would be most appropriate, as there would be too much information to cover in one meeting.

### ii) Evaluation of the process using a SCTU pilot trial

In March 2022, we identified the Cancer Research UK REMoDL-A trial (NCT04546620) as a typical SCTU drug trial which was about to start holding IDMCs [19]. The CI, TMs and statisticians involved were all approached and agreed that as a single arm, randomised drug trial with standardised endpoints (e.g. progression-free survival and overall survival) and no complexities in trial design (e.g. adaptive design, complex interventions), REMoDL-A would be suitable to be involved in the pilot training programme. As we began this pilot, REMoDL-A was already open and recruiting patients. An introductory IDMC meeting had been held, but the first meeting where trial data would be presented had not taken place. Working with the Chief Investigator (CI) and TMs, the PPI coordinator recruited a suitable public contributor for the pilot trial.

We approached a public contributor (Irene Soulsby) who had worked with SCTU on previous projects and was known to be confident at taking on new PPI challenges and open to learning new skills. Importantly, they also had no other links to the trial or trial management group, in order to maintain the independence of the IDMC group. This public contributor then underwent the training programme outlined in Fig. 2.

Following the first IDMC meeting, the public contributor was sent an evaluation questionnaire to gather feedback on the training programme and process of being involved in the IDMC.

Evaluation questionnaires were also sent to the CI, TM, statisticians and IDMC chair to help assess the success of the pilot, any difficulties or problems encountered, and any further work or resources that may be needed to continue and extend the project.

### iii) Refinement of the process

Using the feedback and evaluation from the pilot, some changes were made to the training programme. These included increasing the time for each introductory meeting to allow more time for questions from the public contributor and a list of potential resources and documents that should be sent to the public contributor in advance of these meetings (the revised project plan and training programme can be viewed in Supplementary file 1).

We also updated the evaluation questionnaires used. The public contributor evaluation form used in the pilot was sent to three independent public contributors for review of the wording and relevance of the questions being asked and was updated. The pilot evaluation questionnaires used to gather feedback from the trial CI, statisticians and IDMC chair were also reviewed by the SCTU trial methodologist and updated. The updated questionnaires were used for further evaluation (Supplementary file 2).

### iv) Further evaluate the process in additional SCTU trials

The updated training programme was evaluated in an additional three SCTU trials which were starting or due to have their first IDMC meeting (NERO (NCT05455424), AURORA (NCT05038657) and P+R-ICE (NCT05221645)). N.B. The CI of one further trial did not want a public contributor to be on the IDMC for their trial as they had concerns that the clinical sections of the meeting would be too complicated for a lay member to follow, therefore that trial was not included.

All public contributors were required to sign the IDMC charter and confidentiality agreement for their respective trials and were reimbursed for their time in line with the SCTU PPI payment policy, which follows the NIHR payment guidelines. Reimbursement of public contributors involved in research is widely accepted as best practice in UK research [20] and is generally not considered a conflict of interest for decision making in oversight committees.

### v) Using feedback to finalise the process for future trials

Evaluation questionnaires (see ‘Supplementary file 2 - Evaluation questionnaires, v2 Feb 23’) were sent to the public contributors, CIs, trial managers, statisticians and IDMC chairs after the first IDMC meeting of the three trials.

This feedback was used to refine and finalise the process of including public contributors in IDMCs of future SCTU trials.

## Findings

A total of four trials were included in the evaluation of this pilot study: REMoDL-A, NERO, AURORA and P+R-ICE, all of which have IDMC meetings.

Evaluation took the form of written questionnaires which were emailed to all those involved in the pilot. Data in the public contributor questionnaire was collected through a combination of numbered Likert scale questions and open-ended questions with free-text responses. Data in the questionnaires for CIs, trial managers, statisticians and IDMC chairs was collected through open-ended questions and free-text responses.

Of the four trials for which evaluation was sent out, feedback was received from all four public contributors, four TMs, one trial coordinator, six statisticians, three IDMC chairs, and two CIs (one of whom is CI for both the P+R-ICE and REMoDL-A trials).

We have used a combination of narrative findings and direct quotes from the questionnaire responses to illustrate our findings.

### Introductory training sessions

All four public contributors involved in the pilot rated the introductory sessions with the CI, TMs and statisticians as very satisfactory (5 on a Likert scale of 1–5) and were given space to comment on why they had given these scores, which included:

The information was pitched at the right level for me.

At first it seemed like a lot to take in, but it was helpful during my first IDMC meeting.

A very comprehensive process.

One contributor suggested that it would be useful for a lay Plain English Summary (PES) or copy of the Participant Information Sheet (PIS) to be provided in advance of the meetings, to give more information on the trial from a patient’s point of view. This was echoed in feedback from SCTU staff (TMs and statisticians), as well as suggestions that copies of the training presentations are made available after the meetings for future reference. Other suggestions for resources that should be provided in advance included an FAQ document on IDMCs for public contributors and template agendas and slides for staff delivering the training due to the time taken to prepare the sessions.

One contributor fed back that they had some prior knowledge of graphical interpretation and datasets and commented that someone with less experience may need more training on that aspect. However, feedback from another contributor was:

I had not had a lot of experience with statistics in the past. I had ample time to ask questions and go over things if necessary.

When asked to describe the introductory sessions in three words, the words provided were **helpful**, **informative**, **necessary**, **relevant**, **explanatory**, **encouraging**, and **meaningful**.

Following the pre-IDMC meeting with the statisticians to go through the IDMC data report, feedback received included:

The pre-meet ensured that I had understood what I had prepped. [The statistician] confirmed the points I had pulled out were relevant and the questions I had very relevant. This gave me confidence for the first meeting.

I identified more issues I needed clarifying. I had the confidence to ask questions that were very much from the point of view of a public contributor.

SCTU staff (TMs, trial coordinators and statisticians) were positive in their feedback on the introductory training sessions.

Feedback from the initial evaluation of the first trial included in the pilot (REMoDL-A) suggested that the training sessions should be made longer than originally scheduled to allow more time for questions. This was implemented for the remainder of the pilot, and feedback was that there was ample time for explanation and questions.

Feedback from a CI did point out that a less experienced CI may find it more challenging to talk about the specific roles and responsibilities of the IDMC and that a public contributor taking on this role will require a certain level of learning confidence or previous experience with data to take part.

In some cases, we found it difficult to arrange introductory meetings with the CI for a trial, often down to CIs’ busy diaries and lack of availability. For this reason, the CI for one trial did not take part in the introductory training sessions. In these cases, the TM was able to give a lay overview of the disease area and trial background at the start of their session with the public contributor.

### IDMC meetings

All four public contributors provided positive feedback about their experiences of attending their first IDMC meetings:

I felt well prepared and supported.

I was made to feel welcome and my inputs were acknowledged.

It is a little daunting as a layman… the information from the introductory sessions helped guide me during the meeting.

One public contributor did comment that previous experience of PPI for trials may be of benefit for this particular role:

For me it was fine, but for somebody with less experience of trials then that side of the process might need more information.

All four public contributors continued their roles on the IDMCs following the initial meeting. Their feedback also showed that there is an interest and desire to be more involved in numerical aspects of clinical research, with all four answering “yes” when asked if they would be interested in future PPI roles involving data and statistics. One contributor has since joined another trial IDMC after their role on the first trial ended.

Feedback from SCTU staff who are involved in the IDMC meetings showed that the public contributors involved in the pilot were able to make meaningful contributions to the IDMC meetings, which in some cases directly influenced next steps:

[They] asked questions that provoked discussion, which led to the CTU team querying data that was incorrect.

[They] made a valid contribution to the meeting by querying the sample demographics.

The public contributor made some important contributions and made a suggestion for additional information he would want to see in one table in the future, which the other IDMC members agreed with.

There were five patients who had ethnicity recorded as ‘not known’ and she highlighted the importance of this data. This prompted us to raise queries on the database with sites to find these missing data points.

Feedback from the Chairs of the IDMCs was also gathered after the first meeting, and again was widely positive about the inclusion of public contributors:

The questions did not distract from the discussion but added to it.

One chair did feedback that it is important that public contributors understand not just the advantages, but also the possible disadvantages of a trial. Another commented that having previous experience sitting on a TMG and gaining knowledge of general trial processes would be beneficial for any public contributor considering taking part in an IDMC.

## Conclusions

Through this pilot project we have been able to use qualitative research methodologies, such as design-based research and action research, to develop and refine the training materials required for public contributors to be able to meaningfully take part in IDMCs. Beyond this, the feedback received from public contributors, trial teams and IDMC members suggests that the inclusion of public contributors, when adequate training and resources are provided, is welcomed by those involved in IDMCs and that their input can enhance the work of these committees, as evidenced in direct quotes from open-ended questions in the feedback questionnaires:

Having the PPI present during the meetings allows for a fresh perspective.

[The public contributor] brought a fresh perspective to the meeting, with further insights gained in the process.

I feel that it is important to have a public member giving their opinion and input. Certainly, a valuable addition.

I think it has been successful so far and I would continue/expand our experience of doing this.

While the feedback gathered shows that public contributors did have a meaningful impact on these committees in this pilot, their input mainly centred around provoking discussion and querying information or data provided in the IDMC report. There was no empirical evidence that the decision-making of these committees changed due to the presence of public contributors. However, this was a small study, and further work is needed to fully assess the impact and feasibility of routinely including PPI in IDMCs (see the ‘Limitations to this research and future work’ section below).

**Fig. 3:**
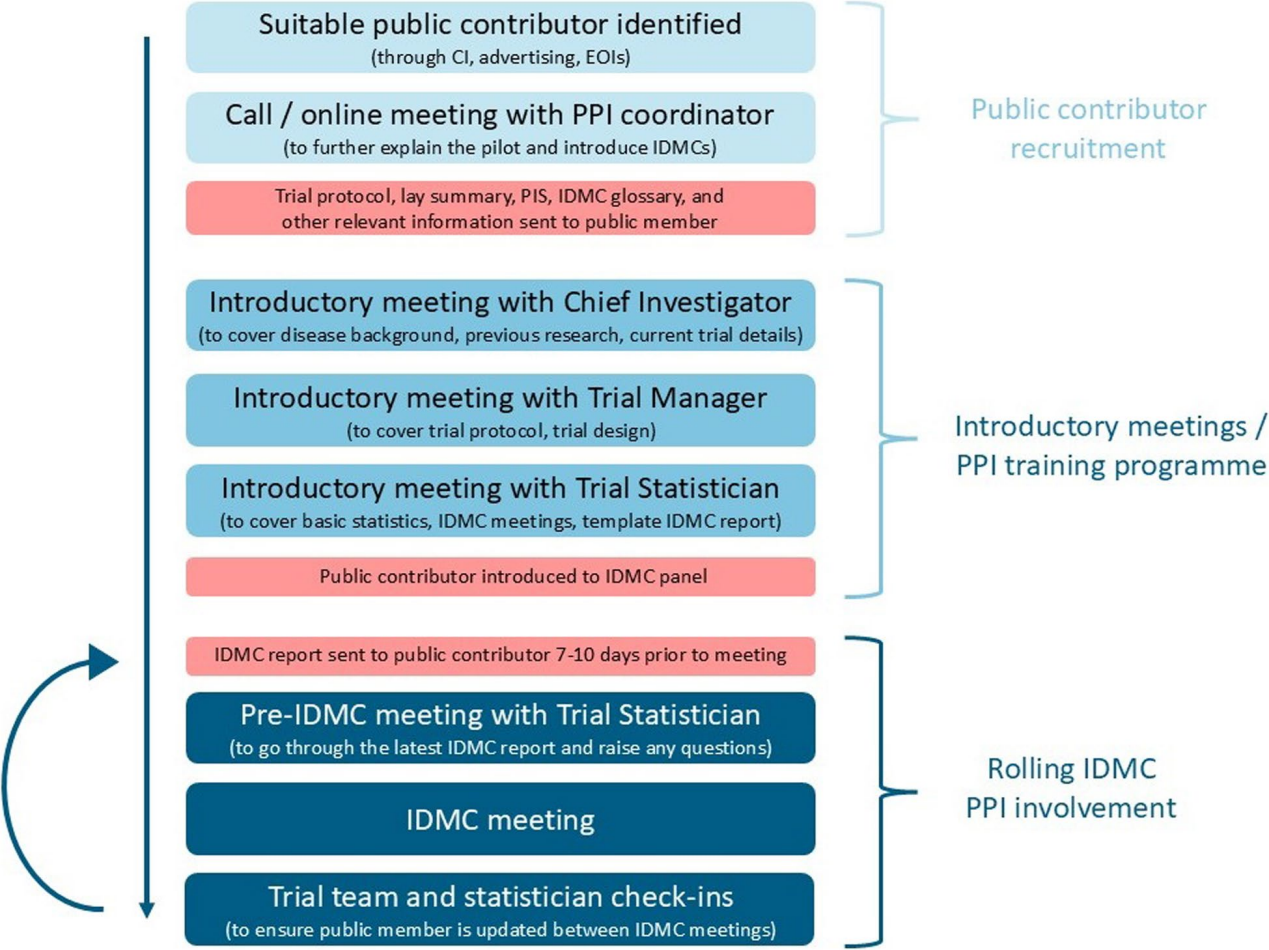
Introductory training programme for public contributors on IDMCs

From the pilot we have reviewed and refined the introduction and training process for public contributors joining an IDMC and developed an introductory training programme (Fig. 3):

For many other trial oversight groups, SCTU routinely includes two or more public contributors on each committee. This is to provide balance between public contributor/lay opinions and feedback, and to provide opportunities for peer support between public contributors. However, IDMCs are very small committees, with the independent members generally comprising of two clinicians and a statistician. Therefore, we suggest having one public contributor on IDMCs would be appropriate, with support and mentoring provided by the trial team.

Other resources that may be needed and should be considered for future expansion of the pilot:

For public contributors:

- A glossary of terms and acronyms frequently used in IDMC meetings.
- An FAQ document answering basic questions about what an IDMC is and does.
- Protocol and PIS/PES for the trial.
- Signposting to online resources or training on basic principles of statistics, e.g. Kaplan-Meier curves, confidence intervals, *p*-values, etc.
- An introduction to the other IDMC panel members—this could be via email and with a supporting document giving short bios on each member.

For trial teams/CIs:

- Background information on the public contributor and their previous PPI experience and lived experience (if happy to provide).
- Template agendas and slide sets for introductory meetings.

For IDMC chairs and members:

- Background on the IDMC pilot project and training provided to public contributors taking part in meetings.
- Background information on the public contributor and their previous PPI experience and lived experience (if happy to provide).
- An introduction to the public contributor—this could be via email.

Through the pilot feedback and evaluation, we also identified several other considerations that may be useful for teams looking to include public contributors on IDMCs and similar statistical/data-driven committees:

- Finding public contributors: Thought needs to be given to how opportunities for IDMCs are advertised to the public to ensure those applying have the necessary skill sets and confidence to cope with the training and complex nature of the meetings. Advertisements should outline the nature of IDMCs and any previous experience or skills needed.
- Experience of public contributor: Initial feedback from statisticians involved in the pilot suggested a public contributor with direct experience of the condition being researched in the trial would be of benefit. For example, one of the public contributors had experience of cancer, but not the cancer being evaluated. However, an interest in data, safety, trial procedures and regulations was also found to be just as important/beneficial to involvement in IDMCs. Feedback from both public contributors and one IDMC chair suggested that public contributors beginning involvement in IDMCs should have some previous experience in PPI in clinical trials, specifically in a role such as a TMG member where they would have gained knowledge and experience of general trial processes and terminology.
- Terminology: When initially speaking to public contributors, we found that terminology and explanations given were very important and could influence whether they wanted to take part. For example, using the term ‘introductory session’ instead of ‘training’ may be less daunting for a public contributor. There is a need to be upfront about the requirements of the role and the IDMC, but acknowledging that the words used can be important.
- Ongoing mentoring: While the above training programme represents necessary formal training for public contributors taking on the IDMC PPI role, more informal mentoring could also be considered from the trial statistician, wider trial team and CTU PPI coordinator to ensure the public contributor has the ongoing support and resources to continue in their role, as is the case for any public contributor taking on a PPI role at a CTU.
- PPI Coordinator: The PPI Coordinator at SCTU was often the initial point of contact for public contributors, and although very experienced in PPI work, has no direct experience of IDMCs or data reports. This may not necessarily be detrimental, as a more lay approach can be reassuring to new public contributors. However, consideration needs to be given to how much information they can provide and whether any additional training is needed for someone fulfilling this role.
- Lack of detailed feedback/insight provided in questionnaires: Responses to emailed questionnaires are not always forthcoming, and some answers were brief or provided little detail. Therefore, there is possibly a need for future qualitative interviews (see the ‘Limitations to this research and future work’ section below)
- Timing of introductory training: Carrying out the training programme well in advance can be detrimental if there are then problems with recruitment that mean IDMC or Safety Review Committee (SRC) meetings are delayed. This was the case for one of the trials initially selected for the pilot to have a public contributor on the SRC, and the trial ended up being excluded from the evaluation. Processes may be needed to look at refresher training sessions if required due to delays.
- Resources: The extra training and support required to include public contributors on IDMCs means this is more resource-intensive than some other PPI for both staff and public contributors. Consideration needs to be given to who takes on these roles and what resources are available, as well as extra budget for PPI reimbursement for more, potentially longer meetings.

### Limitations to this research and future work

The success of this pilot project has led to the expansion of including public contributors on IDMCs at the SCTU. However, this was a pilot project conducted in a small number of trials at one UK CTU, and there are therefore limitations to the scope and findings of the research. Here we outline these limitations and identify possible future work that may help to improve knowledge and processes in this area:

- Resource creation: While the training programme was well received and had a positive impact with public contributors, trial teams and IDMC chairs, additional resources were identified that could help maximise the usefulness and impact of the process. We are now producing the resources mentioned in the conclusion to ensure that public contributors, staff, CIs and IDMC members have adequate information and resources to allow PPI in IDMC meetings.
- Implementation planning: The inclusion of public contributors on IDMCs and the training involved does require extra staff input and time, and this may be a limitation to CTUs’ abilities and willingness to implement this additional PPI role. Further work could look at the resources needed to implement the training programme and how this can be streamlined using template resources (as above) and guidance.
- Qualitative interviews: Feedback for this pilot was collected through questionnaires sent via email, rather than qualitative interviews. This was primarily due to the small nature of the pilot, as well as not wanting to over-burden public contributors and historical difficulty in finding availability with researchers and IDMC chairs. There are therefore limitations to the amount of information collected, with little or no follow-up when information is missing or lacking. We therefore propose to carry out qualitative interviews with those involved to provide further assessment of the pilot project and to expand this qualitative work as the training programme is rolled out more widely across SCTU to continue to assess its impact and feasibility. This may also include feedback from other IDMC members and from the public contributor who is part of a SCTU IDMC that was not included in the pilot project for comparison. This more in-depth work may require additional funding and resources, which the pilot did not have.
- Consideration for other trial designs: This initial piece of work was conducted with the aim to include PPI contributors on cancer clinical trials at SCTU and included single arm and randomised trials of drug interventions where the endpoints were standardised and relatively easy to understand (e.g. progression-free survival and overall survival). Although we can use this training programme for similarly designed interventional trials in any disease area, our SCTU study portfolio also includes more complex biomarker-guided platform trials, large translationally driven observational studies in early diagnosis and complex intervention mixed methodology trials, which may be more challenging for public contributors. So, there is a need to consider if the process should be adapted for public contributors for these different types of study, with potentially more in-depth and longer training to encompass these more complex trial designs and statistical methodologies. SCTU is an academic trials unit, and this project therefore focuses only on academic-led trials. However, we see no reason the training outlined in this project could not be adapted and used within commercial trials.
- Assessing impact: This pilot focussed on the creation and development of the training and resources needed to successfully include PPI in IDMCs. Narrative findings using responses from the evaluation questionnaires did also provide positive insight into the feasibility of including public contributors and their impact on the committees; however, further evidence from future qualitative work is needed to assess the long-term impact and decision-making outcomes for IDMCs routinely adopting PPI.
- Demographic data: Other than gender, we did not collect official demographic data from the public contributors involved in the pilot, or from other members of the trial teams and IDMCs. This should be an additional part of any future qualitative work to ensure inclusion of public contributors on IDMCs is inclusive and that additional requirements or reasonable adjustments are taken into consideration so that everyone is given equitable opportunities to take part.
- Geographical scope: This project was carried out in a UK CTU. As outlined in the Background section, PPI is generally well established in UK clinical research, providing a suitable bedrock for expanding PPI into more complex, numerical areas such as IDMCs. Our findings are therefore hopefully relevant and applicable to other CTUs within the UK. However, there may be differences in how widely PPI is currently embedded and conducted in other countries’ healthcare and regulatory systems, and this may need to be taken into account for international organisations wishing to implement PPI in IDMCs.

## Supplementary Information

Supplementary Material 1. IDMCs PPI pilot project and training plan, v2 Jan 23.

Supplementary Material 2. IDMC pilot evaluation questionnaires, v2 Feb 2023.

## Data Availability

Data sharing is not applicable to this article as no datasets were generated or analysed during the current study. We have added supplementary files for others to access and use/adapt for their own purposes.

## Abbreviations

CI: Chief Investigator
CTU: Clinical trials unit
DMEC: Data Monitoring and Ethics Committee
DMSC: Data Monitoring and Safety Committee
EOI: Expression of interest
FAQ: Frequently asked questions
IDMC: Independent Data Monitoring Committee
PES: Plain English Summary
PIS: Participant Information Sheet
PPI: Patient and public involvement
SCTU: Southampton Clinical Trials Unit
SRC: Safety Review Committee
TM: Trial manager
TMG: Trial management group
TSC: Trial Steering Committee
UKCRC: United Kingdom Clinical Research Collaboration
