# Supplementary material for: Including patient and public contributors on clinical trial Independent Data Monitoring Committees (IDMCs)
Source: Trials. 2026 Feb 17;27:145. doi: 10.1186/s13063-026-09559-w (PMC12914982; doi:10.1186/s13063-026-09559-w)
Supplement: Supplementary file 1 — Supplementary Material 1. IDMCs PPI pilot project and training plan, v2 Jan 23. [file 13063_2026_9559_MOESM1_ESM.docx]

**Patient and Public Involvement on Independent Data Monitoring Committees (IDMCs) - Pilot project plan**

**[Note – IDMCs are also sometimes referred to as Data Monitoring and Ethics Committees or DMECs)**

**Vision:**

To include patient and public involvement (PPI) in all areas of clinical trial design, set-up and governance. To achieve this, and as we expect funders such as Cancer Research UK to start demanding evidence of PPI in all trial oversight groups in the future, the Southampton Clinical Trials Unit (SCTU) is trialling the inclusion of a public contributor on IDMCs.

**Pilot Plan:**

***This plan was updated in Jan 2023 to reflect the feedback from the initial pilot evaluation***

| **Aim:** |
| --- |
| To establish PPI representation in IDMCs for SCTU trials. We will assess the feasibility of including a public contributor in IDMC meetings and establish what training and resources they will need to take part in discussions. |
| **Project Oversight:** |
| The pilot project will be facilitated by the SCTU PPI coordinator, Liz Allaway, along with the trial managers and statisticians for trials involved in the pilot.  Current trials involved:   - REMoDL-A *(pilot underway)* - NERO - AURORA - P+R+ICE |
| **Pilot:** |
| - In March 2022, we identified REMoDL-A as a trial which was about to start holding IDMCs and would be suitable for the pilot. - Working with the trial managers (Nicole Keyworth, Josh Caddy) and senior statisticians (Louise Stanton, Geoff Saunders) the PPI coordinator recruited a suitable public contributor to sit on the IDMC. We discussed the need to have someone who is comfortable looking at data and tables of numbers, and who will be confident to give opinions in a meeting with the CI, statisticians, and independent IDMC members. ***Following evaluation of the initial pilot we also suggest the public contributor*** ***has some lived experience of the disease area, either as a patient or carer/family member.*** - We carried out appropriate training and provide the necessary support for the public contributor to be able to understand and contribute to the IDMC meetings (see below training section) - The public contributor is required to sign the IDMC charter. - The public contributor will be reimbursed for their time at meetings and for carrying out pre-meeting reading and preparations, in line with the NIHR guidelines. - The PPI coordinator may attend the initial IDMC meetings for each trial as an observer to provide support to the public contributor and to gain insight for the pilot project. The coordinator will sign an appropriate charter/NDA if required. - We will carry out an evaluation of the pilot to assess its feasibility, gain feedback from all IDMC members, and plan future work required to take the project forward to IDMCs of other SCTU trials. |
| **Training and resources:** |
| The PPI coordinator held discussions with the trial managers and statisticians for the initial pilot trial (REMoDL-A) on the training and resources needed to help a PPI representative take part and make a meaningful contribution to a IDMC meeting.  ***Following evaluation of the REMoDL-A pilot, we revised this training plan to take into account the feedback received from the public contributor, trial team, Chief Investigator and IDMC chair.***  We have identified a four-step training plan for public contributors on IDMCs:   - An introduction from the Chief Investigator (1 hr): background to the disease area, previous research and the background to the trial. Allow time for the public contributor to ask questions. - An introduction from the Trial Manager (1 hr): to go through the protocol, PIS and any relevant trial materials and make sure the public contributor has a good understanding of the trial procedures and aims. Allow time for the public contributor to ask questions. - An introduction to DMECs from the Stats team (1 hr): including the purpose of the meetings, running through a standard IDMC report, going through relevant terms, acronyms and statistical methodology. Allow time for the public contributor to ask questions. - Pre- IDMC meeting (1 hr): ahead of each IDMC meeting the stats team should meet with the public contributor to go through report that will be discussed at the upcoming meeting, to ensure the public contributor has an understanding of the data and a chance to ask preliminary questions.   Other resources that may be needed:   - A glossary of terms and acronyms frequently used in IDMC meetings. - A Participant Information Sheet (PIS) and/or Plain English Summary (PES). - An introduction to the other IDMC panel members – this could be via email and with a supporting document giving short bios on each member. |
| **Evaluation:** |
| ***Following the REMoDL-A pilot, the PPI coordinator worked with the SCTU qualitative researcher (Cherish Boxall) and three independent public contributors to review and revise the feedback questionnaires used to evaluate the project.***  The PPI coordinator will seek feedback from everyone involved in the pilot project, including:   - The public contributor - Trial Managers - Statisticians - The Chief Investigator/clinician - IDMC chair   This will be used to assess the success of the pilot project, any difficulties or problems encountered that need addressing, and any further work and resources that may be needed to continue and extend the project. |
| **Future work:** |
| Qualitative study:   - There may be the chance to carry out some qualitative assessment of the pilot project by interviewing public contributors and IDMC panel members. - This will require an extension to the project with more trials / IDMCs involved.   ***Update, Jan 23 – potential Case Study:***   - Development of template slides for staff delivering training to public contributors. - Potential publication - Trials journal is running a collection of case studies on IDMCs and best practice. |
