# Supplementary material for: Including patient and public contributors on clinical trial Independent Data Monitoring Committees (IDMCs)
Source: Trials. 2026 Feb 17;27:145. doi: 10.1186/s13063-026-09559-w (PMC12914982; doi:10.1186/s13063-026-09559-w)
Supplement: Supplementary file 2 — Supplementary Material 2. IDMC pilot evaluation questionnaires, v2 Feb 2023. [file 13063_2026_9559_MOESM2_ESM.docx]

**Independent Data Monitoring Committee (IDMC) - PPI pilot project evaluation - Public Contributors**

1) Was the role well explained to you before agreeing to take part in any training or meetings?

**Yes No**

*(either highlight your chosen option or delete the other option)*

If you answered no, what further information could have been provided?

2) How did you find the introductory session with the Chief Investigator *[insert CI name]*, with 1 being very unsatisfactory and 5 being very satisfactory?

1 2 3 4 5

One two three four five

*(either highlight your chosen number or delete the other numbers)*

Is there anything that could have made this session better? Was the level and amount of information provided easy to understand?

3) How did you find the introductory session with the Trial Manager *[insert TM name]* with 1 being very unsatisfactory and 5 being very satisfactory?

1 2 3 4 5

One two three four five

Is there anything that could have made this session better? Was the level and amount of information provided easy to understand?

4) How did you find the introductory session with the Statistics team *[insert MS name]* with 1 being very unsatisfactory and 5 being very satisfactory?

1 2 3 4 5

One two three four five

Is there anything that could have made this session better? Was the level and amount of information provided easy to understand?

5) Did the pre-meeting session with the statistics team to go over the IDMC report provide you with everything you needed to take part in the IDMC?

6) How would you describe the introductory sessions in three words?

7) How did you find the process of giving feedback at your first IDMC meeting?

8) Is there anything else we could have done to help you feel more prepared and able to take part in the IDMC?

9) Based on your experience of our training programme, would you be interested in participating in future research PPI roles involving data/statistics if asked?

**Thank you for completing this feedback questionnaire. If filling in an electronic document, please send your responses via email to** [**L.Allaway@soton.ac.uk**](mailto:L.Allaway@soton.ac.uk)**. If filling in a paper version, please use the pre-addressed envelope provided.**

**Independent Data Monitoring Committee (IDMC) - PPI pilot project evaluation – Trial Team and Chief Investigator**

1) How did you find your training session with the public contributor for *[insert name of trial]*?

2) What could be done to make the process easier for the public contributor?

3) What could be done to make the process easier for those delivering training?

4) *[For stats only]* How did you find the meeting with the public contributor to go through the IDMC report ahead of the first IDMC meeting?

5) *[For stats only]* What impact did the public contributor’s comments have on the IDMC report/ pre-IDMC meeting?

5b) Would you rather receive feedback on the report from the public contributor via email or in the meeting? Why?

6) What other considerations or measures do you think we need to put in place to assist public contributors who are attending future IDMC meetings?

**Thank you for completing this feedback questionnaire. Please send your responses via email to** [**L.Allaway@soton.ac.uk**](mailto:L.Allaway@soton.ac.uk)**.**

**Independent Data Monitoring Committee (IDMC) - PPI pilot project evaluation - IDMC members**

1) Were you aware of a public contributor being included on the *[insert trial name]* IDMC ahead of their first meeting?

2) How did you find having a public contributor in the IDMC?

3) How did the comments/questions raised by the public contributor add to/distract from the IDMC discussions?

4) The public contributor had training with the Chief Investigator, trial manager and statisticians ahead of the IDMC meeting as part of the SCTU IDMC PPI Pilot Project and will continue to receive support from the SCTU team going forward.

What considerations or measures should we put in place to assist public members joining IDMCs in the future?

**Thank you for completing this feedback questionnaire. Please send your responses via email to** [L.Allaway@soton.ac.uk](mailto:L.Allaway@soton.ac.uk)**.**
